# Peptide Inhibitors of Insulin Fibrillation: Current and Future Challenges

**DOI:** 10.3390/ijms24021306

**Published:** 2023-01-09

**Authors:** Beatrice Rosetti, Silvia Marchesan

**Affiliations:** Chemical and Pharmaceutical Sciences Department, University of Trieste, 34127 Trieste, Italy

**Keywords:** insulin, peptides, fibrillation, amyloid, chirality, D-amino acids, diabetes, inhibitors, β-sheets, phenylalanine

## Abstract

Amyloidoses include a large variety of local and systemic diseases that share the common feature of protein unfolding or refolding into amyloid fibrils. The most studied amyloids are those directly involved in neurodegenerative diseases, while others, such as those formed by insulin, are surprisingly far less studied. Insulin is a very important polypeptide that plays a variety of biological roles and, first and foremost, is at the basis of the therapy of diabetic patients. It is well-known that it can form fibrils at the site of injection, leading to inflammation and immune response, in addition to other side effects. In this concise review, we analyze the current knowledge on insulin fibrillation, with a focus on the development of peptide-based inhibitors, which are promising candidates for their biocompatibility but still pose challenges to their effective use in therapy.

## 1. Introduction

### 1.1. Pathological Amyloidoses

The uncontrolled growth of pathological amyloid fibrils remains today an unsolved general challenge, and its inhibition is very relevant to the medical and pharmaceutical fields. Protein aggregation leading to the formation of amyloid fibrils has various adverse effects on human health, and there are more than 30 proteins or peptides that have been identified as amyloidogenic in humans. The deposition of amyloids can affect one or more organs, such as the heart, liver, and kidneys, leading to crucial dysfunction [1]. Some of these pathologies are Alzheimer’s, Huntington’s, and Parkinson’s diseases, diabetes type 2, amyotrophic lateral sclerosis, frontotemporal dementia, insulin-derived amyloidosis, and spongiform encephalopathies. Each of these diseases is associated with fibrils of a specific protein, such as amyloid β, huntingtin, α-synuclein, insulin, and many others [2]. Different amyloid proteins can interact with each other [3] and even promote further fibrillation through cross-seeding events, whereby stable nuclei (i.e., seeds) of one protein can favor the aggregation of another [4].

The ability of these proteins or peptides to form amyloids is not a generic property of the primary structure, because they do not have significant amino acid sequence homology, although they form aggregates that share the same cross β-sheet pattern, with β-strands arranged perpendicular to the fibrillar axis, stabilized by hydrogen bonds [5]. Three major factors that can lead to the conversion of the partially or totally unfolded state of a protein into amyloid fibrils were identified: high hydrophobicity, high propensity to convert from an α-helix toward a β-sheet, and low net charge [6]. Usually, aggregation involves proteins that are either unfolded or folded into a nonfunctional structural state, and the molecular mechanisms of amyloid fibrillation in vivo are complex and not yet fully elucidated [7]. Disordered and dynamic regions of proteins, in particular, have been suggested to play an important role in the process. They can either drive unfolding and aggregation through the exposure of hydrophobic regions that are otherwise buried in the protein core, or expose amino acid sequences for proteolytic cleavage that may result in insoluble fragments prone to fibrillation [8].

Amyloids are formed by normally soluble proteins, which assemble into insoluble fibrils that are generally considered resistant to protease-mediated degradation. Recent evidence showed that in vitro amyloids can be enzymatically hydrolyzed [9], while ex vivo amyloids are more resistant, suggesting that in vivo they are selected by proteases, in the sense that only those that are not cleared by hydrolysis endure and accumulate [10].

The structure of fibrils is thermodynamically very stable [11], but the traditional view that the process is completely irreversible has been challenged, as evidence is mounting that shows otherwise [12]. In particular, one hypothesis that has been advanced in the past is that amyloids could serve as a long-term deposit, from which functional proteins could be recovered when needed, in an analogy to what happens for physiological hormonal storage in granules [13]. This concept had been proposed for the sustained release of insulin to treat diabetes [14]. However, insulin fibrils can elicit inflammation and cytotoxicity [15]. Insulin amyloids can promote the fibrillation of other proteins too, such as amylin [16]. Furthermore, insulin dysfunction has been linked directly or indirectly to increased risks of developing neurodegenerative syndromes [17]. Nevertheless, there is increasing evidence that amyloids may also play a physiological role, although this is far from being understood for human amyloids, while a clearer scenario has emerged for other organisms, such as bacteria [18]. Despite the many roles that insulin fibrillation may play, pertaining to various facets of human health, it is not as widely studied as other amyloid proteins, although it clearly deserves further investigation.

### 1.2. Insulin Structure

**Figure 1:**
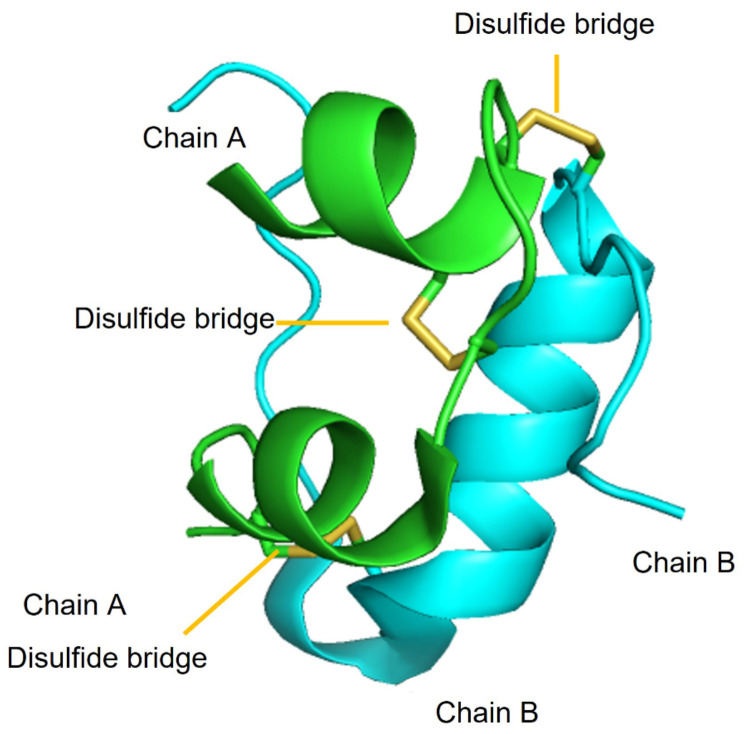
The three-dimensional structure of insulin shows three helical portions, two of chain A (green) and one of chain B (cyan), and three disulfide bridges (yellow).

The primary sequence of insulin was elucidated in a landmark work led by Sanger and Smith [19]. Insulin is a highly conserved protein (~5.8 kDa) secreted by the pancreatic β-cells of the islets of Langerhans. It is a heterodimer composed of two polypeptides called chain A and chain B, consisting of 21 and 30 amino acids, respectively. The two chains are connected together by three disulfide bridges: two of them between chains A and B (Cys7_A_-Cys7_B_ and Cys20_A_-Cys19_B_), and the other one within chain A (Cys6-Cys11). These six cysteine residues are responsible for holding together the tertiary structure with three α-helices (Figure 1) that are conserved across all members of the insulin superfamily. The disulfide presence still poses challenges to the high-yielding chemical synthesis of insulin, which nowadays is mainly produced as recombinant protein through biotechnological tools [20].

Human insulin is stored as a hexamer, comprising three dimers held together through the coordination with two zinc ions. The hexamer dissociates in dimers and monomers. These two forms are more prone to aggregation, but the monomer is also the physiologically active form [21].

### 1.3. Insulin Biogenesis and Storage in Granules

Insulin is produced by β-cells that are located within the islets of Langerhans in the pancreas. Insulin is stored in large granules with a dense core, from which it is released in response to increased glucose levels in the blood. Its secretion is controlled at the β-cell level, as glucose uptake and metabolism produces ATP that causes the closure of ATP-sensitive K^+^ channels, leading to membrane depolarization and the subsequent opening of voltage-dependent Ca^2+^ channels, an influx of Ca^2+^ ions, and exocytosis of insulin granules [22]. 

At the cell level, insulin is produced as preproinsulin, which is transported to the endoplasmic reticulum (ER) thanks to an N-terminal signal sequence, which is then cleaved off as the peptide is translocated across the organelle membrane. The resulting proinsulin is a single polypeptide that folds assisted by chaperones, including glucose-regulated protein 94 (GRP94), and it forms the three native disulfide bonds. In the Golgi apparatus, proinsulin is stored in granules together with other bioactive peptides. There, the maturation of the hormone includes several steps that are not yet fully elucidated, including the cleavage between residues 31 and 64 of proinsulin to release the C-peptide and yield insulin, which then is zinc-coordinated into hexamers and forms aggregates promoted by the presence of Ca^2+^ ions [22].

### 1.4. Insulin Function

During meals, some carbohydrates are used immediately, and some are stored as glycogen in the liver and muscle cells. Insulin plays an important role in this process because it is necessary to transport glucose in the cells for use or storage. Insulin facilitates the uptake of food components into the cells, and also their storage because it suppresses the enzyme that breaks down glycogen and fat. This is a vital process for correct metabolism. Indeed, the absence of insulin creates a catabolic state, in which the liver produces new glucose, resulting in hyperglycemia in diabetic patients [23]. Insulin controls glucose levels in the blood, maintaining glucose homeostasis because it facilitates the monosaccharide diffusion in adipose and muscle cells through the modulation of the translocation of the glucose transporter GLUT4 [24]. This protein also plays a role in lipid metabolism in fat tissue and the liver [25]. Furthermore, insulin can act as a ligand for insulin-like growth factor receptors, which are involved in cell proliferation and growth in physiological and pathological conditions [26]. Several downstream signaling molecules activated by insulin are located in the brain, and it has been suggested that insulin may play a role also in other types of amyloidoses [27]. Therefore, it is clear that insulin has multiple biological activities that are involved in a variety of processes linked to human health. First and foremost, insulin is a crucial hormone to treat diabetes, as discussed more in detail in the following section.

### 1.5. Insulin Therapy for Diabetes

In 2021, there were 537 million diabetes patients worldwide, and that number is expected to grow to 783 million by 2045 [28]. Diabetes can be mainly classified into two categories: type 1 and type 2. The first one, also called “insulin-dependent diabetes”, is due to auto-immune destruction of the pancreatic β-cells that produce insulin, leading to its deficiency, and it accounts for just 5–10% of diabetic patients [29]. Type 1 diabetes is associated with a higher risk for cardiovascular disease, blindness, and nerve and kidney damage, and for these reasons, the control of blood glucose levels is a treatment priority [30]. Type 2, called also “non-insulin-dependent diabetes”, accounts for 90–95% of all diabetic patients. The causes for poor glucose control in these patients are diverse, and they are often related to excessive weight and insufficient physical activity. Individuals with insulin resistance or insulin deficiency are included in type 2 too. These patients may not need insulin treatment to survive [29], but 30.8% need insulin therapy [31]. To control insulin levels, there are two main methods: multiple daily injections (with pens or syringes), or continuous subcutaneous insulin infusion with the use of a pump, which can better mimic physiological insulin release, as the dose infused can be adjusted as needed [32].

The gold standard insulin therapy is the “basal bolus” regimen, which aims at mimicking the physiological insulin production by the pancreatic cells. It combines long-acting and fast-acting insulin types through multiple daily injections. The long-acting form is usually injected at bedtime and provides the basal level of insulin, while the rapid form is injected to counteract the post-prandial glucose peaks, as it has a rapid onset and shorter duration [33]. The different forms are attained through different approaches. The long-lasting form typically stabilizes the hexamer and/or provides a supramolecular formulation of insulin, for instance through complexation with zinc or protamine. Another strategy involves the attachment of a long-chain fatty acid to prolong the binding of serum albumin to insulin. Conversely, the fast-acting insulin is in the monomeric form, readily available, and is usually obtained through mutations that destabilize β-sheet interactions between monomers. One example is the “lispro” insulin, where Pro28_B_ and Lys29_B_ are inverted [20]. 

## 2. Insulin Fibrillation

Studies on insulin fibrillation started in the early years of the 20th century and it is still an important research topic. Insulin fibrillation is one of the biggest problems for diabetic patients. Subcutaneous insulin injection may cause several types of injection site-related lesions, such as lipohypertrophy, lipoatrophy, and insulin-derived amyloidosis [34]. The latter is a specific form of localized cutaneous amyloidosis composed of insulin-fibril deposits at the site of insulin injection [35,36]. This mass is also called an “insulin ball”, and it causes poor blood glycemic control due to inadequate absorption of insulin and a reduced amount of physiologically active monomer; so the diagnosis of this disease becomes critical in diabetic patients [37,38]. Furthermore, this amyloid deposit may cause fouling of the infusion-pump system and lead to reduced or erratic delivery rates, with poor penetration of the injected insulin, and variable biological response to it [39]. For instance, a study compared the levels of serum insulin obtained with insulin injection into amyloid deposits, with those achieved after injection into another point of the skin, and the absorption at the nodules was a remarkable 34% of the other [37]. It is thus not surprising that diabetic patients are instructed to change the sites of insulin injections.

**Figure 2:**
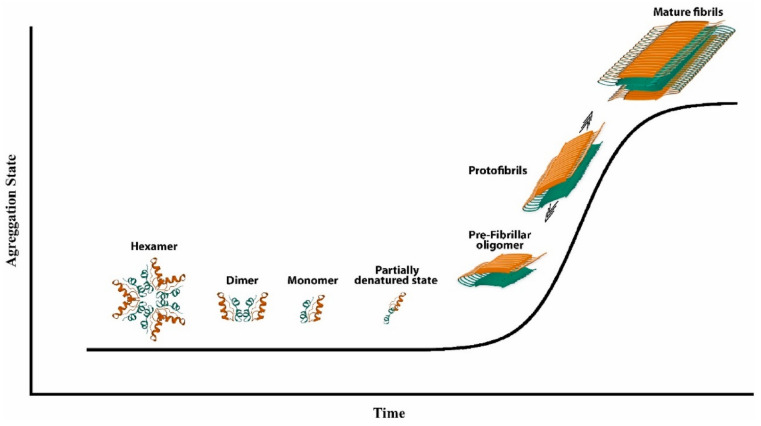
Scheme of insulin fibrillization. Reprinted from [21] with permission from Elsevier, Copyright © 2023 Elsevier.

To understand insulin fibrillation, it is important to consider the various forms of insulin and how they convert into each other. At physiological pH, insulin is present in its zinc-bound hexameric form, which is mostly helical (see Figure 1 above), while the monomer is less conformationally stable [40]. As a monomer, insulin can undergo partial unfolding to expose hydrophobic regions of the peptide that can lead to aggregation (Figure 2) [41]. Monomeric and dimeric forms are more prevalent at acidic pH, whereby functional groups are protonated and cannot coordinate zinc ions. Other factors that accelerate insulin fibrillation include heating, mechanical agitation, and high ionic strength. All these conditions can promote the exposure of the hydrophobic surfaces, while ions can screen the charges and enable hydrophobic interactions [42]. Shifting the equilibrium to the hexameric form could be an idea to avoid insulin fibrillation, but this approach would also bear disadvantages, considering that insulin is active in the monomeric form. 

The mechanism which leads insulin to form fibrils is a physical process that follows a sigmoidal curve, with a lag phase, rapid growth, and a plateau (Figure 2). The mechanism of insulin’s fibril formation has been proposed to consist of three main reactions: nucleation, growth, and precipitation [43]. Nucleation consists of the formation of stable nuclei, and it is viewed as the assembly process toward an organized structure. Subsequent additions of monomers to the nuclei proceed to form the fibrils [44]. However, the experimentally observed kinetics are more complex and suggest the formation of secondary nuclei, as well as fibrils’ fragmentation and branching [42]. Interestingly, the disulfide bridges do not undergo rearrangement during fibrillation and remain intact [45].

**Figure 3:**
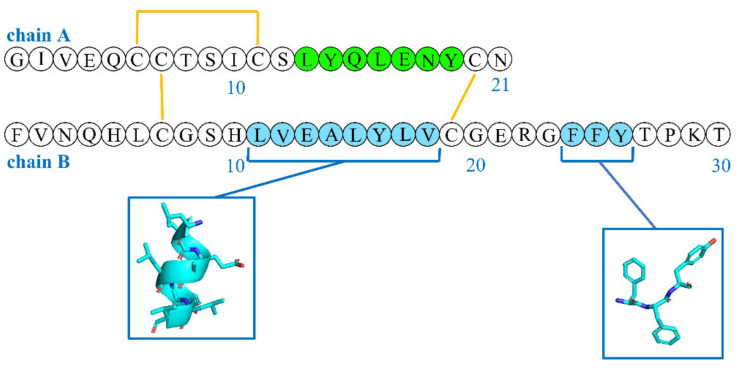
Sequences of insulin chains A and B with colors (green for chain A and cyan for chain B) highlighting hydrophobic regions that can be exposed and promote fibrillation.

Hydrophobic interactions are probably the driving force of the aggregation, because a critical step in the formation of the fibrils is the exposure of the hydrophobic regions [44]. Fibrils feature β-sheets, whose spline was hypothesized to arise from the hydrophobic sequences LYQLENY from chain A (green, Figure 3) and LVEALYL from chain B (cyan in Figure 3) [46]. Another, less studied, hydrophobic region that plays a key role in the formation of β-sheets includes the “aromatic triplet” FFY, which is located at the C-terminus of chain B [47]. This aromatic triplet is also a part of the β-strand region and supports antiparallel β-sheet formation [48]. However, this region is also important in mediating the binding with the insulin receptor [49], and mini-insulins that are devoid of this motif compensate for it through the inclusion of other tyrosine residues via specific mutations (e.g., G20Y in chain B) [50]. Interestingly, the peptide sequence FFY is conserved in various self-antigens of autoimmune diabetes, and its administration was shown to prevent the onset of type 1 diabetes in mice models [51].

Insulin has a high propensity to aggregate and it forms amyloid deposits in patients with type II diabetes both with continuous subcutaneous insulin infusion and with repeated insulin injection [44]. The fibrillation of insulin poses a variety of problems in its production, storage, and delivery. An interesting fact is that insulin does not form amyloid fibrils when it is normally produced in the body, but it does in industrial production, purification, or transport because most of these steps require low pH and agitation [44,47].

### 2.1. Effect of Agitation on Insulin Fibrillation

Some experiments indicated that, at 37 °C and without vigorous agitation, insulin did not fibrillate, while it did with mechanical agitation. In one experiment, the effect of the concentration of insulin on the kinetics of aggregation was monitored at 960 rpm of agitation, with no significant differences as a function of concentration, ionic strength, or pH [44]. Insulin aggregates’ growth rate was recently found to be proportional to shear stress under agitation, with the insulin aggregation rate correlating with the amount of dynamic triple interfaces, which arise from the contact between air–liquid interfaces with solid surfaces. In these conditions, the meniscus’s continuous movement during agitation leads to the generation of transiently dry areas, causing the partial dehydration of adsorbed proteins that may then aggregate more easily [52]. Similarly, fibrillation is promoted by mechanical shocks and cavitation, which can occur when pressure waves of sufficient amplitude produce gas bubbles within liquids that rapidly expand and collapse, generating localized regions of high energy dissipation [43]. Finally, a recent study showed that increasing interfacial shear rate produces a monotonic increase in the fibrillation rate, with a monotonic decrease in fibrillation time [53]. Clearly, these phenomena are very relevant to the industrial production and transport of insulin, and they may be relevant also for infusion pumps that are in contact with the body of diabetic patients during intense physical activity.

### 2.2. Effect of Low pH on Insulin Fibrillation

At 37 °C and acidic pH, there is a decrease in the lag phase, and an increase in the rate with which fibrils grow, relative to what happens at physiological pH values; although, in the pH range from 1.6 to 3.0, there is actually an increase in the lag time and a decrease in the rate of fibril growth [44,54]. Certain preservatives, such as methylparaben, can undergo hydrolysis and generate acidic species, such as 4-hydroxybenzoic acid [55], which can lower the pH of insulin formulations and promote fibrillation [42]. In the case of infusion pumps, the pH may become acidic as a result of carbon dioxide mixing or leaching of tubing components [56]. However, it is unlikely that these factors can reach the extremely acidic pH used in many experiments (i.e., pH < 3), and the relevance of such conditions used in vitro to those found in vivo is an important topic that deserves further investigation.

### 2.3. Effect of Insulin Concentration on Fibrillation

The increase in insulin concentration influences the kinetics of aggregation. In particular, it results in a decrease in the lag time, and in an increase in the rate with which fibrils grow [44]. In 2021, the minimum gelling concentration was found for insulin to correspond to 2.35 ± 0.27 mg/mL, with gels arising from a network of fibrils [53]. However, fibrils can obviously also form at lower concentrations, depending on other factors, such as pH, temperature, agitation, and so on. In particular, it is the monomer concentration that is a critical factor in fibrillation, in agreement with the importance of the nucleation stage in the process [47]. The potential role of supersaturation conditions in amyloid fibrillation has been recently discussed [57]. In vivo, the site of repeated insulin injection is clearly the area with a higher risk for fibrillation promoted by a high local concentration of the protein.

### 2.4. Effect of High Ionic Strength on Insulin Fibrillation

The lag time and apparent rate constants for insulin aggregation were studied in presence of increasing concentrations of various sodium salts, such as sodium chloride, sodium sulfate, and sodium phosphate. The results indicated that an increase in high ionic strength decreased the lag time [44]. Anions have also been studied for their influence on insulin fibrillation, including chloride and sulfate. In particular, the presence of sulfate above 5 mM led to a salting-out effect, with insulin precipitation into aggregates with a high content of α-helix, delaying their conversion into the fibril-forming β-sheets, relative to chloride anions [58]. Furthermore, at acidic pH, at which insulin is present as a polycation, electrolytes play a key role in screening insulin charges and in enabling the hydrophobic interactions leading to fibrillation [59]. Clearly, the issue of high ionic strength promoting insulin fibrillation is more relevant to industrial production, storage, and transport of insulin than to physiological conditions where ion homeostasis is tightly controlled.

## 3. Peptide Inhibitors of Insulin Fibrillation

Insulin is one of the best model proteins to study amyloid fibrillation and its inhibition thanks to its low cost, solubility at relatively high concentration, and commercial availability [60]. Furthermore, the number of insulin fibrillation inhibitors is very low relative to other amyloids, thus warranting the scope for further investigation. In the literature, various classes of nanomaterials and molecules have been considered to inhibit insulin fibrillation. Nanomaterials offer unique properties that arise from working at the nanoscale and are often used in medicine to provide a qualitative leap in therapy [61]. Among nanoparticles (NPs), those studied against insulin fibrillation include silicon NPs [62], ceria NPs [63], magnetite NPs [64], gold NPs [65], silver NPs [66], graphene oxide [67], organic NPs [68], carbon dots [69,70,71], and quantum dots [72]. Among molecules, there are glycopolymers [73,74], amphiphiles [75], aromatic molecules [76], polyphenols [6,77,78], vitamins [79], dyes [80], and various drugs [81]. The idea of the inhibition of amyloid aggregation, especially for insulin, is that molecules with hydrophobic functionalities can sequester the aggregation-prone regions of the target protein and inhibit its aggregation, while exposing more hydrophilic groups to assist with solubility. These interactions can decrease the rate of fibrils’ growth or increase the lag phase of fibrillation. However, it is not easy to predict the consequences or the inhibitory effects of inhibitors. 

**Figure 4:**
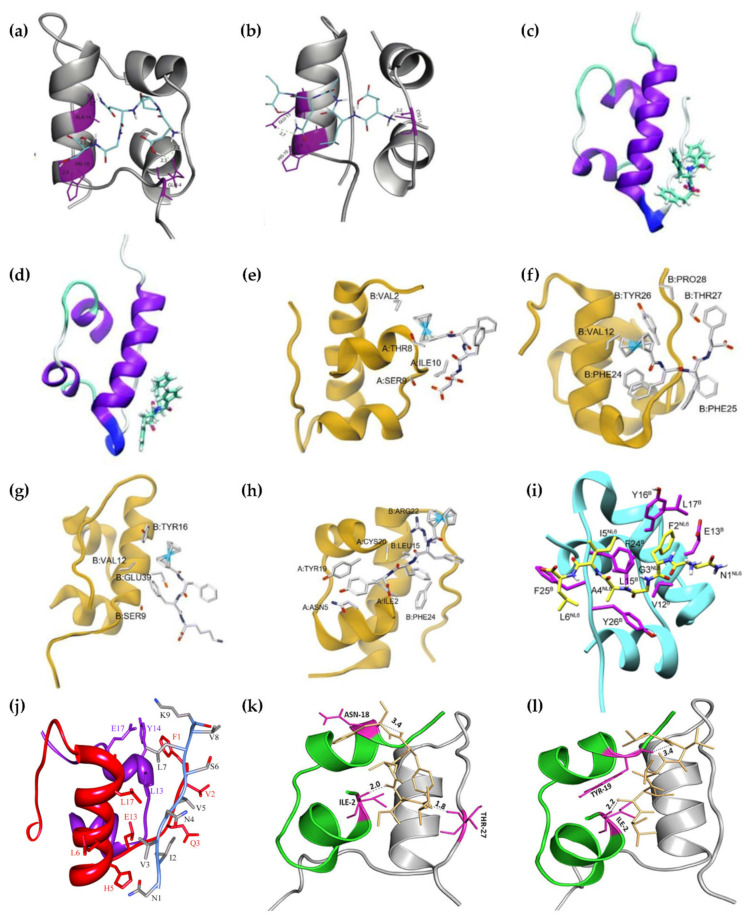
Molecular models of insulin interacting with different peptide inhibitors. (**a**) DNPQS. (**b**) ELAQM. Both are reprinted from [87], Copyright © 2023, with permission from Elsevier. (**c**) Ferrocenyl-FF. (**d**) Ferrocenyl-FY. Both are reprinted from [88], with permission from the Royal Society of Chemistry. (**e**) Ferrocenyl-FFD. (**f**) Ferrocenyl-FFF. (**g**) Ferrocenyl-FFK. (**h**) Ferrocenyl-FFY. (**e**–**h**) They are reprinted from [89], with permission from the Royal Society of Chemistry. (**i**) NFGAIL. Reprinted from [91], Copyright © 2023, with permission from Elsevier. (**j**) NIVNVSLVK. Reproduced from [92] under a Creative Commons license, © 2023 Banerjee et al. (**k**) VIFYW. (**l**) VVVVV. (**k**,**l**) Both are reprinted from [93] under a Creative Commons license, © 2023 Siddiqi, Alam, Iqbal, Majid, Malik, Nusrat, Alam, Ajmal, Uversky, and Khan.

A particular class of inhibitors that is particularly promising in light of its inherent biocompatibility and specificity of action is that of peptides. However, peptide therapeutics are not devoid of drawbacks. Potential pitfalls include limited bioavailability, especially due to limited absorption or rapid enzymatic hydrolysis [82], and risks of an immune response, especially with long-term therapies [83]. Fortunately, several approaches are proving successful in overcoming these limitations, including innovative formulations, chemical modifications, and virtual screenings [82]. Peptide inhibitors (Table 1) have been designed in various ways, often taking inspiration from known protein–protein interactions, e.g., exploiting known sequences that interact with insulin, for instance inspired by the insulin receptor, or by regions of insulin itself that are prone to self-aggregation. A figure that shows the regions of insulin interacting with peptide inhibitors is Figure 4. Clearly, there are different strategies that can be employed. One consists of stabilizing the prefibrillar state to avoid elongation into fibrils, and it was recently found that there are physiologically occurring compounds that can do so effectively, such as fibrinogen [84]. Other approaches include the development of strong binders of protein monomers, or species that can cap the ends of the growing fibril to block its elongation, or agents that absorb onto the surface of oligomers to prevent nucleation [85]. 

### 3.1. AF- and FA-Perylenebisimides

The two isomers studied to inhibit insulin fibrillation were based on the different positioning of alanine and phenylalanine in dipeptides, which were conjugated to perylene units. The design was based on a perylene core, to establish interactions with the hydrophobic regions of insulin, and the polycyclic unit was derivatized at both ends with dipeptides to increase its aqueous solubility. Both isomers acted at the initial stages of aggregation, causing an increase in the lag phase. This is one of the very few studies that successfully reproduced insulin fibrillation in vitro at conditions similar to those found in vivo, i.e., pH 7.4 and 37 °C [86].

### 3.2. DPNGS and ELAQM

The two pentapeptides adopted β-sheet and α-helical conformations, respectively. They were designed by choosing amino acids that featured frequently in the two secondary conformations in various proteins. ELAQM engaged in H-bonding with the residues C11 of insulin chain A, and amino acids H10 and E13 of chain B. DPNGS established H-bonds with Q4, H10, and A14 of chain B. As a result of these interactions, ALMQQ inhibited insulin structural transition from α-helix to β-sheet, while DPNGS reduced the ability of insulin to engage in self-associated β-sheets, leading in both cases to a reduction in fibril-associated fluorescence in the Thioflavin T assay by ~85%. This study required the use of pH 2.0 and heating to 65 °C for 72 h to successfully reproduce insulin fibrillation in vitro [87].

### 3.3. Ferrocenyl Peptides

The FF motif is well-known for being derived from the amyloid β peptide associated with Alzheimer’s disease [96] and it had been identified in 2003 as the minimalistic sequence of the peptide that featured a strong propensity toward self-association [97]. It has been used to develop fibrillation inhibitors of amyloid sequences that feature FF themselves, most notably amyloid β, especially by elongating the sequence with β-breaker amino acids, such as proline [98,99]. In 2020, FF was thus conjugated to ferrocene and shown to be able to inhibit insulin fibrillation, alongside its variant FY [88]. In 2022, the FF sequence was elongated with a third variable amino acid, and was shown to engage in non-covalent interactions with different amino acids of insulin, as shown in Figure 4. In particular, ferrocenyl-FFF and ferrocenyl-FFY proved to be the best inhibitors [89]. Both studies required the use of acidic conditions (i.e., pH 2.0) and heating to 60 °C over several days to reproduce insulin fibrillation in vitro [88,89].

### 3.4. FVPRK

The sequence was inspired by the insulin receptor, which contains the sequence FVPR in its C-terminal portion added to lysine to favor solubility. The peptide was able to coat insulin to yield NPs that could provide a means for the sustained release of active insulin over time. The driving force for the binding to the target appeared to be based on π–π interactions between the aromatic triplet of the C-terminus of insulin chain B, and the phenylalanine of the peptide. As a result of such interaction, in vitro assays showed inhibition of fibrils’ growth, with a delay by a factor of six in the rising of fluorescence, as measured with the Thioflavin T assay. Moreover, the growth rate was slower and the maximum fluorescence obtained with the peptide accounted for ~30% relative to the insulin control. This study successfully reproduced insulin fibrillation in vitro at physiological conditions (i.e., pH 7.4 and 37 °C) after removing any zinc that could promote insulin folding into hexamers by chelation with ethylenediaminetetraacetic acid (EDTA) [90].

### 3.5. KPWWPRR

This heptapeptide was derived from the antimicrobial peptide indolicidin, which is another inhibitor of insulin aggregation. This sequence was based on a core PWWP motif with both tryptophan and proline residues that are known to act as β-breakers, flanked by ionizable amino acids that consisted of lysine and arginine residues. Interestingly, however, the mechanism of action differed from that of many other inhibitors, including those featuring W repeats, with no significant effect on the initial lag phase. It appeared instead that the peptide reduced the rate of fibrillar growth and its amount by acting at a later stage of fibrillar elongation. The residues involved in the binding were found to correspond to the hydrophobic sequence ^11^LVEALYL^17^, and also G20, E21, F24, and Y26, especially with the tryptophan residues of the inhibitor, through hydrophobic and electrostatic interactions. This study required an acidic pH of 2.6 and heating to ~62 °C (335 K) to successfully fibrillate insulin in vitro [39]. 

### 3.6. LVEALYL

This sequence was identified by Ivanova et al. as being responsible for insulin fibrillation. In particular, it was shown that two copies of this sequence could engage in steric zippers [46], which establish dry regions that exclude water and provide great stability to amyloids [100], and in some cases, can include aromatic residues in the so-called phenylalanine zippers [101,102]. It was found that LVEALYL both nucleated and inhibited the fibrillation of insulin at pH 2.5, depending on the molar ratio, with the strongest inhibition observed when it was used in an equimolar manner relative to insulin [46]. 

### 3.7. NFGAIL and NFGAXL

The sequence NFGAIL is a fragment of the human islet amyloid polypeptide (hIAPP) or amylin, which is stored in pancreatic cells together with insulin. Both can form amyloids, and in particular, the sequence NFGAIL is responsible for the fibrillation of amylin. It was thus hypothesized that it could interact with insulin. Other analogs were prepared with the addition of known β-breakers, such as proline, α-aminoisobutyric acid, or α,β-deydrophenylalanine. However, the parent peptide NFGAIL displayed the best performance in terms of reducing the rate of fibrillation and increasing the lag phase, overall leading to fewer fibrils at pH 2.6. The main interactions with insulin occurred through van der Waals force and face-to-face π–π interactions between the F of NFGAIL and the Y16 of insulin chain B, and hydrophobic interactions between the I of the hexapeptide and the FF of the FFY triplet of insulin, as well as H-bonding between the N of the inhibitor and the L17 of insulin chain B [91].

### 3.8. NIVNVSLVK 

This nonapeptide was derived from the SARS coronavirus E-protein primary structure. It contained several hydrophobic amino acids, which could establish weak interactions with those of insulin, effectively delaying its fibrillation at pH 2.6 and 62 °C, i.e., increasing the lag time. This peptide had an effect on the self-association process in the pre-fibrillar nucleation phase, while it did not influence fibrillar elongation. The residues of insulin that could bind the nonapeptide were identified as F1, V2, N3, L6, H10, and L17 of chain B, and L13, Y14, and D17 of chain A. As a consequence of these interactions, the residues H5 and N3 of the chain B of insulin could engage in a salt bridge or H-bond. Finally, K9 of the nonapeptide could engage in H-bonding too, with Y14 of the chain A of insulin [92].

### 3.9. VIFYW and VVVVV

Both peptides are hydrophobic and adopted β-sheet conformations. Both agents could effectively slow down the fibrillar elongation and reduced the amounts of mature fibrils at pH 2.0 and 60 °C, with VIFYW being more effective than VVVVV. The better performance of the former sequence was attributed to the presence of aromatic residues, which could interact with those of insulin. Importantly, both sequences proved effective in disaggregating preformed insulin fibrils [93]. 

### 3.10. VYYR

This tetrapeptide was selected from a screening of a library consisting of prion protein-derived tetrapeptides. Despite being such a short sequence, it proved effective in inhibiting insulin fibrillation at pH 2.6 and 37 °C over a month or 62 °C over several hours, and in maintaining insulin monomers and dimers in a biologically active state. It was found that VYYR is bound to the N-terminal region of the chain B of insulin through a triad H-bonding [94]. 

### 3.11. W_n_-Taurine (n = 1–4) Conjugates

Four different peptide conjugates were designed based on the attachment of 1–4 tryptophan residues to taurine, to provide asymmetric or symmetric inhibitors. The compounds were designed to exploit the ability of tryptophan to engage in several non-covalent bonds, including aromatic interactions and H-bonding, specifically with the aromatic triplet FFY featured at the C-terminus of the chain B. Inhibition of the nucleation phase and retardation of the fibrils’ elongation step were observed at pH 1.6 and 65 °C [95].

### 3.12. Techniques to Study Inhibition of Fibrillation

A good approach to studying the inhibition effect on insulin fibrillation is to use three types of techniques, as shown in many studies that feature in Table 1. Ideally, the three different types should include spectroscopic, microscopic, and calorimetric methods. This is particularly important because indirect assays, such as those based on Thioflavin T fluorescence, can also lead to false positives or false negatives; thus, they should be performed with appropriate controls and the results should be confirmed with other non-spectroscopic techniques. The most popular spectroscopic techniques include circular dichroism (CD) to assess protein conformation [103] and Thioflavin T fluorescence, which is the most popular amyloid dye [104]. Other methods include infrared spectroscopy, nuclear magnetic resonance spectroscopy, or other types of fluorescence based on dyes or the intrinsic fluorescence of amino acids, especially tryptophan [105]. Clearly, the vast majority of these methods are indirect, and in any case, they do not provide information on the morphology of the aggregates, which is best assessed by microscopy techniques. In some cases, Thioflavin T itself has been used as a fluorescent marker for confocal microscopy, but ideally, electron microscopies (especially methods based on transmissions, such as TEM and cryoTEM) provide images with sufficient resolution to analyze, in detail, fibrils’ morphology [106]. Other types, such as atomic force microscopy (AFM) [107], are sometimes used, especially to measure fibrils’ diameter. Finally, calorimetric techniques, such as isothermal titration calorimetry (ITC) and differential scanning calorimetry (DSC), provide useful information on the stability of fibrils and the amount of energy required for their transition to other phases [108]. It should be noted, however, that ITC and DSC are very different techniques that do not provide the same type of information. In particular, ITC provides a direct quantification of the amount of heat that is absorbed or released during the association of biomolecules (such as protein–peptide) in solution to measure the interaction affinity [109]. In particular, the binding constants of the two perylene bisimides described in Section 3.1 were 1.92 × 10^4^ ± 148 and 2.79 × 10^3^ ± 119 M^−1^ [86], and that of VYYR (Section 3.11) was calculated as 3.46 × 10^−3^ M^−1^ [94]. However, it is not always possible to generate sufficient amounts of heat exchange to derive binding affinities [91], even though ITC is a sensitive technique.

## 4. Conclusions

Inhibition of amyloid aggregation is a very important field of research to find a therapy for a large number of diseases. Insulin is vital for some diabetic patients; therefore, its fibrillation is a serious concern. This process still needs to be studied to improve strategies for effective inhibition. In particular, it would be useful to understand which step of the mechanism that ends with fibril formation is the best to inhibit, and by which type of approach, for instance in terms of which form of insulin should be ideally targeted, and which sequence in particular and how. Indeed, while it is important to keep the biologically-active monomer stabilized, inhibitors should not interfere with its binding with insulin receptors.

Peptide-based inhibitors have great potential and they could be a good choice to improve the quality of life of people suffering from insulin-derived amyloidoses. Peptides could be modified to refine the hydrophobic interactions, electrostatic interactions, hydrogen bonds, or π–π stacking and, therefore, increase their inhibitory effects. Peptide inhibitors were already studied and they will be studied in the future too, thanks to their advantageous properties and their versatility. It would be necessary to find a good combination of motifs that can lead to a very important inhibition, potentially leading to new therapies. Peptides are enjoying a positive era for their development as therapeutics [110,111], with many of the historical barriers for such use being overcome, such as their stability in vivo. Indeed, several approaches have been developed to increase their half-life, such as cyclization [112]. Furthermore, peptides and their nanostructures could also offer new means for the improved delivery of therapeutics [113,114]. The ideal inhibitor of insulin aggregation should not interfere with insulin biological activity, including its interactions with insulin receptors and other biomolecules in vivo, and it should be devoid of side effects. 

In addition to the development of small molecule inhibitors of amyloid fibrillation, several approaches are being investigated to improve the stability of insulin formulations, including the use of variants, glycosylation, active surfaces, additives, and so on [115]. One of the remaining challenges is that although infusion pumps are becoming more popular, they do have drawbacks. Among these, the fact that the insulin contained in these devices is under continuous agitation due to the movements of the human body, potentially promoting fibrillation and occlusion of the pump. The use of active surfaces in these devices could perhaps offer potential solutions to minimize this issue.

Other modern issues pertain to our exposure to pollution and its detrimental effects on our health. For instance, micro- and nano-plastics have entered the food chain, and evidence suggests they can enter cells and alter the native state of physiological proteins, potentially playing a role in the pathogenesis of amyloids too [116]. Clearly, today’s challenges are different from those of a few decades ago, and they should be taken into consideration for the timely progress in the field of innovative insulin therapies.

## Figures and Tables

**Table 1:** Peptide inhibitors of insulin fibrillation.

Sequence	Data Type	Interaction	Ref.
AF-perylenebisimide	Atomic force microscopy (AFM) Circular dichroism (CD) Isothermal titration calorimetry (ITC) Thioflavin T fluorescence Zeta (ξ) potential measurement	Not known	[86]
DPNGS	ANS fluorescence Circular dichroism Molecular modeling Thioflavin T fluorescence Transmission electron microscopy (TEM)	Q4_B_, H10_B_, A14_B_	[87]
ELAQM	ANS fluorescence Circular dichroism Molecular modeling Thioflavin T fluorescence Transmission electron microscopy	H10_B_ and E13_B_ C11_A_	[87]
FA-perylenebisimide	Atomic force microscopy (AFM) Circular dichroism Isothermal titration calorimetry Thioflavin T fluorescence Zeta (ξ) potential measurement	Not known	[86]
Ferrocenyl-FX (X = F, Y)	Circular dichroism Dynamic light scattering (DLS) Molecular modeling Thioflavin T fluorescence Transmission electron microscopy	Chain B	[88]
Ferrocenyl-FFX (X = D, F, K, Y)	Atomic force microscopy Circular dichroism Dynamic light scattering Molecular modeling Thioflavin T fluorescence	I2_A_, N5_A_, YC_A_, RGFFY	[89]
FVPRK	Dynamic light scattering Molecular modeling Thioflavin T fluorescence Transmission electron microscopy	FFY	[90]
KPWWPRR	Atomic force microscopy Circular dichroism Confocal microscopy Molecular modeling Nuclear magnetic resonance (NMR) Thioflavin T fluorescence Transmission electron microscopy Tryptophan fluorescence anisotropy	E13_B_, YL, GEFFY	[39]
LVEALYL	Electron microscopy (TEM/STEM) Molecular modeling Thioflavin T fluorescence Single-crystal X-ray diffraction (XRD)	LVEALYL LYQLENY	[46]
NFGAIL NFGAXL (X = 2-aminobenzoic acid)	Atomic force microscopy Circular dichroism Isothermal titration calorimetry Molecular modeling Nuclear magnetic resonance Raman spectroscopy Thioflavin T fluorescence Transmission electron microscopy	EAL, L17_B_, E21_B_, G23_B_, F25_B_; G8_B_, V12_B_, Y16_B_, V18_B_, R22_B_, Y26_B_; IV_A_, C7_A_, A8_A_, L16_A_	[91]
NIVNVSLVK	Circular dichroism Dynamic light scattering Immunoblotting Infrared spectroscopy Scanning electron microscopy (SEM) Size exclusion chromatography (SEC) Thioflavin T fluorescence Transmission electron microscopy Turbidity	LY_A_, and E17_A_; FVN_B_, L6_B_, H10_B_ and L17_B_	[92]
VIFYW	Acrylamide quenching fluorescence ANS fluorescence Circular dichroism Dynamic light scattering Molecular modeling Thioflavin T fluorescence Transmission electron microscopy	GIVEQ, NY, FYTPK	[93]
VVVVV	Acrylamide quenching fluorescence ANS fluorescence Circular dichroism Dynamic light scattering Molecular modeling Thioflavin T fluorescence Transmission electron microscopy	GIVE, YCN, L15_B_, YTP	[93]
VYYR	Atomic force microscopy Circular dichroism Dynamic light scattering Immunoblotting Isothermal titration calorimetry Molecular modeling Nuclear magnetic resonance Thioflavin T fluorescence	NQH	[94]
W_n_-taurine (*n* = 1–4)	Atomic force microscopy Circular dichroism Molecular modeling Nuclear magnetic resonance Thioflavin T fluorescence	FFY	[95]

## Data Availability

Not applicable.
